# Impact of Insecticide Resistance on the Effectiveness of Pyrethroid-Based Malaria Vectors Control Tools in Benin: Decreased Toxicity and Repellent Effect

**DOI:** 10.1371/journal.pone.0145207

**Published:** 2015-12-16

**Authors:** Fiacre R. Agossa, Virgile Gnanguenon, Rodrigue Anagonou, Roseric Azondekon, Nazaire Aïzoun, Arthur Sovi, Frédéric Oké-Agbo, Michel Sèzonlin, Martin C. Akogbéto

**Affiliations:** 1 Centre de Recherche Entomologique de Cotonou (CREC), Cotonou, Benin; 2 Laboratoire Evolution, Biodiversité des Arthropodes et Assainissement, FAST—UAC, Abomey-Calavi, Bénin; Stanford University, UNITED STATES

## Abstract

Since the first evidence of pyrethroids resistance in 1999 in Benin, mutations have rapidly increased in mosquitoes and it is now difficult to design a study including a control area where malaria vectors are fully susceptible. Few studies have assessed the after effect of resistance on the success of pyrethroid based prevention methods in mosquito populations. We therefore assessed the impact of resistance on the effectiveness of pyrethroids based indoor residual spraying (IRS) in semi-field conditions and long lasting insecticidal nets (LLINs) in laboratory conditions. The results observed showed low repulsion and low toxicity of pyrethroids compounds in the test populations. The toxicity of pyrethroids used in IRS was significantly low with *An*. *gambiae s*.*l* (< 46%) but high for other predominant species such as *Mansonia africana* (93% to 97%). There were significant differences in terms of the repellent effect expressed as exophily and deterrence compared to the untreated huts (P<0.001). Furthermore, mortality was 23.71% for OlyseNet® and 39.06% for PermaNet®. However, with laboratory susceptible “Kisumu”, mortality was 100% for both nets suggesting a resistance within the wild mosquito populations. Thus treatment with pyrethroids at World Health Organization recommended dose will not be effective at reducing malaria in the coming years. Therefore it is necessary to study how insecticide resistance decreases the efficacy of particular pyrethroids used in pyrethroid-based vector control so that a targeted approach can be adopted.

## Introduction

Malaria remains a serious public health burden despite continued control strategies in endemic regions [1]. Indeed, the widespread occurrence of vectors that are resistant to compounds approved by WHO have seriously threatened the previous progress made in malaria vector control over the last decade [2, 3, 4, 5]. In Benin, treated mosquito nets and indoor insecticide spraying are the main methods adopted for malaria control. The principle of these malaria prevention methods rely specifically on killing or repelling mosquitoes that may blood feed on humans. Among the insecticides used, pyrethroids are the only compounds licensed for bed nets and other netting materials used for personal protection. Several studies have shown a spread of Dichloro-Diphenyl-Trichloroethane (DDT) and Pyrethroids resistance characterized by a high level of knock down resistance (Kdr) [5, 6]. Several studies have shown the influence of Kdr on the efficacy of treated nets [7, 8], and there exists evidence of several resistance mutation in various regions [9, 10, 11].

According to Killeen *et al*. [12], direct personal protection of individuals and households is achieved by repelling or kill mosquitoes before they can feed. Pyrethroids showed a strong toxic and repellent effect, so house spraying and treated nets often repel as many mosquitoes as they kill [13]. Another study conducted by N’Guessan et al. [7] which used experimental huts, showed a reduced effect of lambdacyhalothrin treated nets against wild resistant *An*. *gambiae s*.*l* in Ladji which is a suburban area of Cotonou. These wild mosquitoes were not susceptible to the repellent effect of Long Lasting Insecticidal Net (LLINs) and thus have the ability to penetrate treated bed nets and to blood feed, unlike susceptible mosquitoes [14].

A recent study conducted in Benin failed to associate the reduced efficacy of LLINs with an increase in vector resistance to insecticides [8]. One of the limitations of the study was the selection of two similar clusters: one in which mosquitoes were to exhibit resistance and the other in which the mosquito population was to be susceptible. These clusters may be difficult to find in areas where significant resistance is present.

The main question that the Benin National Malaria Control Program is confronted with is whether pyrethroids should continue to be used for malaria vector control without a better understanding of the relationship between resistance and the effectiveness treated materials give that there exists significant resistance to pyethroids in the mosquito populations. It is likely that before 2020 no new compounds will become available that can be used for malaria vector control [15] and it is therefore important to study the link between resistance and the efficacy of pyrethroids to better develop management strategies in the form of a targeted approach in the selection of specific pyrethroids for particular mosquito populations.

## Materials and Methods

### Ethical clearance

Approval (007) was obtained from the ethic committees of the Ministry of Health of Benin. Each trial participant provided written or oral informed consent and was offered chemoprophylaxis during and one month after the experimental hut trial.

### Study site

The study was conducted in both field and laboratory. In the field, four experimental huts at the station of Malanville (11°52’ N and 3°23’ E) in northern Benin were used. The station, built by the Centre of Research in Entomology of Cotonou (CREC) for WHOPES phase II studies, situated by the Niger River. Malanville is characterized by a Soudanian climate (semiarid) and experiences only one rainy season each year with typically a 900 mm rainfall [16]. The *Anopheles gambiae s*.*l* populations in Malanville are resistant to pyrethroids [6]

The huts are designed to simulate local households, following WHO guidelines for laboratory and field testing [17]. The huts are typical of the West African region which are made from concrete blocks with a corrugated iron roof, have a ceiling made of thick polyethylene sheeting and have a concrete foundation slab surrounded by a water-filled channel which is used to prevent ants from entering the structure [16]. Mosquitoes however can easily enter through four window slits. These are made from metal which is fixed at an angle against the wall forming a funnel, such that the bottom of the funnel has a large opening and narrows towards the top where a centimeter wide slit in the outside wall exists. During each evaluation the window slits are opened from 6:00 PM to 5:00 AM by a supervisor. Mosquitoes can fly upward through the funnel to enter through the gap in the wall and into the hut and downwards to exit. This restricts the exit of mosquitoes from the hut and allows for a more accurate count of mosquitoes entering the hut. A single veranda trap projecting from the back wall of each hut is also part of the design and is made of polyethylene sheeting and screening mesh measuring 3m long, 2.5m wide and 1.5m high [16]. The movement of mosquitoes into the huts and veranda is therefore unimpeded during the night.

### Susceptibility testing

These tests were carried out prior to the semi-field experiments. *Anopheles gambiae s*.*l* larvae and pupae were collected in Malanville and reared in the CREC’s insectarium until the adult stage. WHO susceptibility tests were performed using *An*. *gambiae s*.*l* females aged 2–5 days in the following conditions: exposure and holding temperature: 25 ± 2°C, exposure and holding relative humidity: 80 ± 10%. The mosauitoes were exposed to Bendiocarb 0.1%, Deltamethrin 0.05%, Permethrin 0.75%, Fenitrothion 1.0% and Pirimiphos Methyl 0.25% treated papers. After one hour of exposure followed by an hour of observation, the mosquitoes were held in a WHO observation tubes and provided with a 5% honey solution. The following day, mortality was recorded according to WHO protocol [18].

A CDC bottle bioassay was designed in order to evaluate the biochemical resistance in the *Anopheles gambiae s*.*l* population of Malanville. CDC bottles were coated with deltamethrin (12.5 ug/bottle) following CDC guidelines [19]. At least 20 unfed female mosquitoes aged 2–5 days were introduced into four 250 ml Wheaton bottles coated with insecticide and one control bottle coated with acetone only. The number of dead and alive mosquitoes was monitored at 15, 30, 35, 40, 45, 60, 75, 90, 105, 120 minutes intervals.

### Semi-field study

Over a period of six months, the toxicity and repellency of Lambda-Cyhalothrin, Deltamethrin and Alpha-cypermethrin were evaluated in experimental huts in Malanville. The insecticides used per labelled hut at the WHO recommended concentrations were:

Hut 1: REVIVAL 10 WP 10%: (pyrethroid: lambda-cyhalothrin); (0.03g/ m^2^)
Hut 2: Untreated control
Hut 3: PALI 250 WG, 25%: (pyrethroid: deltamethrin); (0.025g/ m^2^)
Hut 4: RUBI 50 WP, 5%: (pyrethroid: alpha-cypermethrin); (0.03g/ m^2^)
N.B: The absorbency of the wall was 124 mL/m^2^.

Indoor Residual treatments were applied using a hand-operated compression sprayer. Only the interior cement walls were sprayed. These walls were sprayed uniformly and only after masking the veranda and window slits with protective coverings. The evaluation started 48 hours after treatment.

The insecticides were tested against host-seeking, wild *An*. *gambiae s*.*l* mosquitoes. The four volunteers who slept in the experimental huts were rotated randomly among huts throughout the four nights of each weekly study using the Latin square. Each volunteer provided written informed consent and was offered chemoprophylaxis during and for one month after the experimental hut trial in which they participated. The consent of each participant was recorded on the form that had been approved by the ethical committee. The volunteers entered the huts at 9:00 PM and slept in until dawn which was at 06:30 AM. They did not leave the huts throughout this period [16] and so were provided portable toilet facilities. In the morning, dead mosquitoes were collected from the floor of the huts using forceps; while resting mosquitoes were collected from the walls, the roofs of the huts and exit traps using mouth aspirators. For each compartment of the huts the numbers of female mosquitoes which were dead or alive, fed or unfed was recorded and morphologically identified using identification keys. All female mosquitoes species collected alive were placed in holding plastic netted cups and fed with a 5% honey solution for 24 hours so as to assess delayed mortality. The entomological effect of treatments was compared to the control for deterrence, induced exophily and blood-feeding inhibition.

Deterrence is a measure of the reduction in hut entry relative to the control huts. Induced exophily is the proportion of mosquitoes that exit the huts and are thus found in exit traps. Blood-feeding inhibition describes the reduction in blood feeding compared with that in the control huts. The number of blood fed mosquitoes (Personal protection) relative to the control hut was calculated as follows: % personal protection = 100(B_u_-B_t_)/B_u_ where B_u_ is the number of blood fed mosquitoes in the untreated control hut and B_t_, the number of blood fed mosquitoes in the huts with insecticide treatments.

### Quantitative bioassays in laboratory

Wild *Anopheles gambiae s*.*l* larvae and pupae were collected from Malanville and reared in the insectarium until reaching the adult stage. The use of *An gambiae* Kisumu helped to assess the efficacy of pyrethroid treated nets PermaNet® 2.0 (Vestergaard Frandsen SA) and OlyseNet® [(Sumitomo Chemicals, Osaka, Japan). PermaNet® 2.0 is a LLIN made of multifilament polyester (75–100 denier) fabric. It is coated with a wash-resistant formulation of deltamethrin at a target dose of 1.8 g/kg (55 mg/m^2^). OlyseNet® is a LLIN made of knitted polyethylene (> 150 denier) thread with permethrin at 20 g/kg (2% w/w) incorporated into the polyethylene fibers during the manufacturing process. Bioassays were done according to WHO procedures for cone tests [19] and therefore two cones were placed on the net. At least five females of the laboratory susceptible *An*. *gambiae* s.s Kisumu strain and wild type *An*. *gambiae s*.*l* resistant from Malanville per cone were exposed to pieces of OlyseNet® and PermaNet® 2.0 (25cm x 25cm) and to untreated nets for 3 minutes [20]. After exposure, the mosquitoes were held for 24 hours while being fed on a 5% honey solution. Bioassays were replicated eight times with a total of 70 to 80 mosquitoes tested for each type of net. Mosquitoes exposed to untreated nets were used as a control. Bioassays were carried out at 25 ± 2°C and 80 ± 10% relative humidity. Knock down and mortality rates were recorded 60 minutes and 24 hours post exposure respectively.

Thirty to forty Kisumu strain adult mosquitoes aged between 5 to 8 days were released at 6:30 PM in compartment A of a 60-centimeters (cm) tunnel (25 x 25 cm square section) made of glass [20]. This compartment had a chamber that was made using LLIN which had been pierced to allow mosquitoes to pass through it into the tunnel. A guinea pig was placed in compartment B. The two compartments were connected through the tunnel. Mosquitoes were allowed to move through the tunnel into the chamber holding the guinea pig. The guinea pigs were housed at CREC and cared for in accordance with the principles for the humane treatment of animals [21] and so each guinea pig was used only once for this study. The following morning at 7 AM, mosquitoes were collected from both compartments and placed in plastic holding cups. Their mortality rate and the feeding status of mosquitoes (blood fed or not) were recorded. Data obtained during the tunnel test with the LLINs (25 x 25 cm) was compared to the control. Measurements of LLIN efficacy included deterrence, blood-feeding inhibition, immediate and delayed mortality rates.

### Statistical analysis

The number of mosquitoes of each species penetrating the huts, the proportion of mosquitoes that deterred early, the proportion of dead mosquitoes within the huts and the proportion of blood fed mosquitoes were compared by species. Quantitative data was analyzed using Poisson regression and logistic regression for proportional data (STATA 12 Software). Time-response curves were fitted to data by using a logistic regression model using R software (http://www.r-project.org/).

## Results

### Susceptibility test

**Fig 1:**
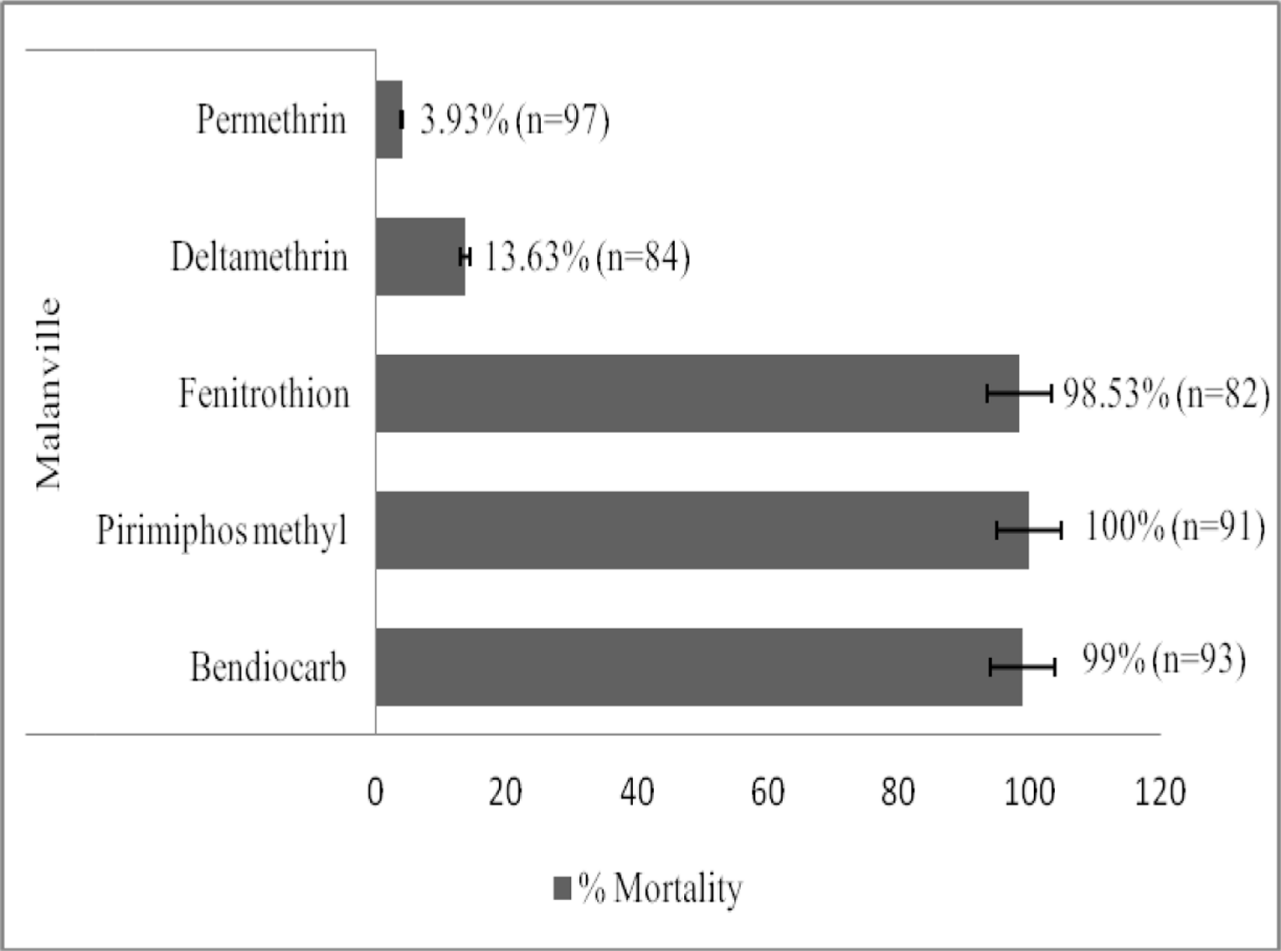
Percentage of dead *An*.*gambiae s*.*l* observed after one hour exposure to papers treated with various insecticides in Malanville. n = number of mosquito tested. Bendiocarb 0.1%, deltamethrin 0.05%, permethrin 0.75%, fenitrothion 1.0% and pirimiphos methyl 0.25% were used.

**Fig 2:**
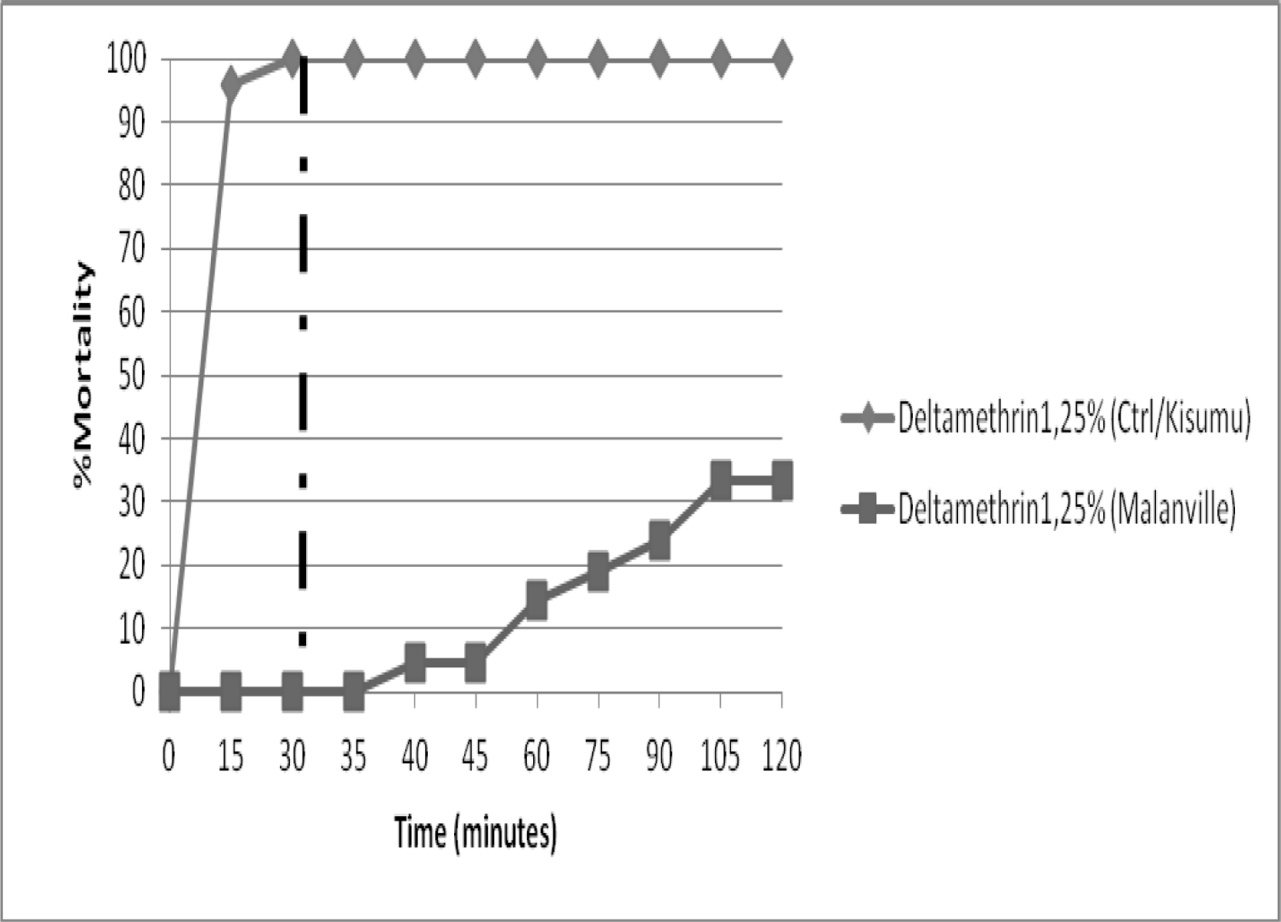
Percentage of dead *An*. *gambiae s*.*l* observed after two hours exposure to CDC bottles treated with deltamethrin 12,5ug/bottle in Malanville.

The susceptibility of *Anopheles gambiae s*.*l* varied between insecticides (Fig 1). In total, 542 female *An*. *gambiae s*.*l* aged from 2 to 5 days were exposed to several insecticides (Fig 1). The mortality rate with deltamethrin and permethrin was 13.63% and 3.93% respectively suggesting resistance to these pyrethroids. With pirimiphos methyl, fenitrothion and bendiocarb, mortality ranged between 98 and 100% suggesting full susceptibility to these insecticides. For the CDC susceptibility test (Fig 2), the mortality rate for deltamethrin was 100% with the laboratory susceptible strain whereas the Malanville population showed resistant with a 0% mortality rate at diagnostic time of 30 minutes.

### Experimental hut trial

#### Repellent effect

A total of 1987 *Anopheles gambiae s*.*l*, 350 *Mansonia africana*, 11 *An*. *pharoensis*, and 20 *Culex nebulosus* were collected during the semi-field study period in Malanville (Table 1). Induced exophily, deterrence and blood feeding inhibition of pyrethroids are summarised in Table 1. Overall exophily, blood-feeding and deterrence rates fluctuated one month to the next over the six-month observation period (S1 Table). All evaluated pyrethroids induced an exophily rate under 37% (Untreated hut: 11.55%; alpha-cypermethrin: 33.64%; deltamethrin: 36.31% and lambda-cyhalothrin 31.71%). Deterency was under 29% (alpha-cypermethrin: 22.28%; deltamethrin: 21.95% and lambda-cyhalothrin 27.89%). There were significant differences in terms of exophily and deterency between the treated huts and the untreated huts (P<0.001). Whether the huts were treated or not did not have any difference on the blood feeding of mosquitoes in the experimental huts (Untreated hut: 84.98%; alpha-cypermethrin: 83.98%; deltamethrin: 86.84% and lambda-cyhalothrin: 80.76%).

**Table 1:** Semi-field parameters measures to assess the repellent effect of alphacypermethrin, deltamethrin and lambda-cyhalothrin in Malanville.

Treatments	Total	Proportion (%)	CI-95%	OR/IDR	CI-95%(OR/IDR)	P
**Exophily**						
CONTROL	606	11.55^a^	[09.01–14.10]	1.00	-	-
Alpha cypermethrin	437	33.64^b^	[29.21–38.07]	3.88	[2.82–5.34]	< 0.001
Deltamethrin	471	36.31^b^	[31.96–40.65]	4.36	[3.19–5.96]	< 0.001
Lambdacyhalothrin	473	31.71^b^	[27.52–35.91]	3.56	[2.59–4.88]	< 0.001
**Blood feeding**						
CONTROL	606	84.98^a^	[82.14–87.83]	1.00	-	-
Alpha cypermethrin	437	83.98^a^	[80.54–87.42]	0.93	[0.66–1.30]	0.659
Deltamethrin	471	86.84^a^	[83.78–89.89]	1.17	[0.82–1.65]	0.388
Lambdacyhalothrin	473	80.76^a^	[77.21–84.31]	0.74	[0.54–1.02]	0.067
**Deterency** *						
CONTROL	606	0.00^a^	[00.00–00.00]	1.00	-	-
Alpha cypermethrin	437	22.28^b^	[18.52–26.04]	0.78	[0.69–0.88]	< 0.001
Deltamethrin	471	21.95^b^	[18.22–25.68]	0.78	[0.69–0.88]	< 0.001
Lambdacyhalothrin	473	27.89^b^	[23.68–32.09]	0.72	[0.64–0.82]	< 0.001

*Regarding deterrence, IDR have been calculated, but for others parameters odds ratio (OR) have been calculated.

‘-‘represents reference rate

For each parameter, the letter a means that there are no difference between untreated hut and the treated huts and the letter b means that there are significant difference between untreated hut and treated huts.

#### Toxicity effect

**Fig 3:**
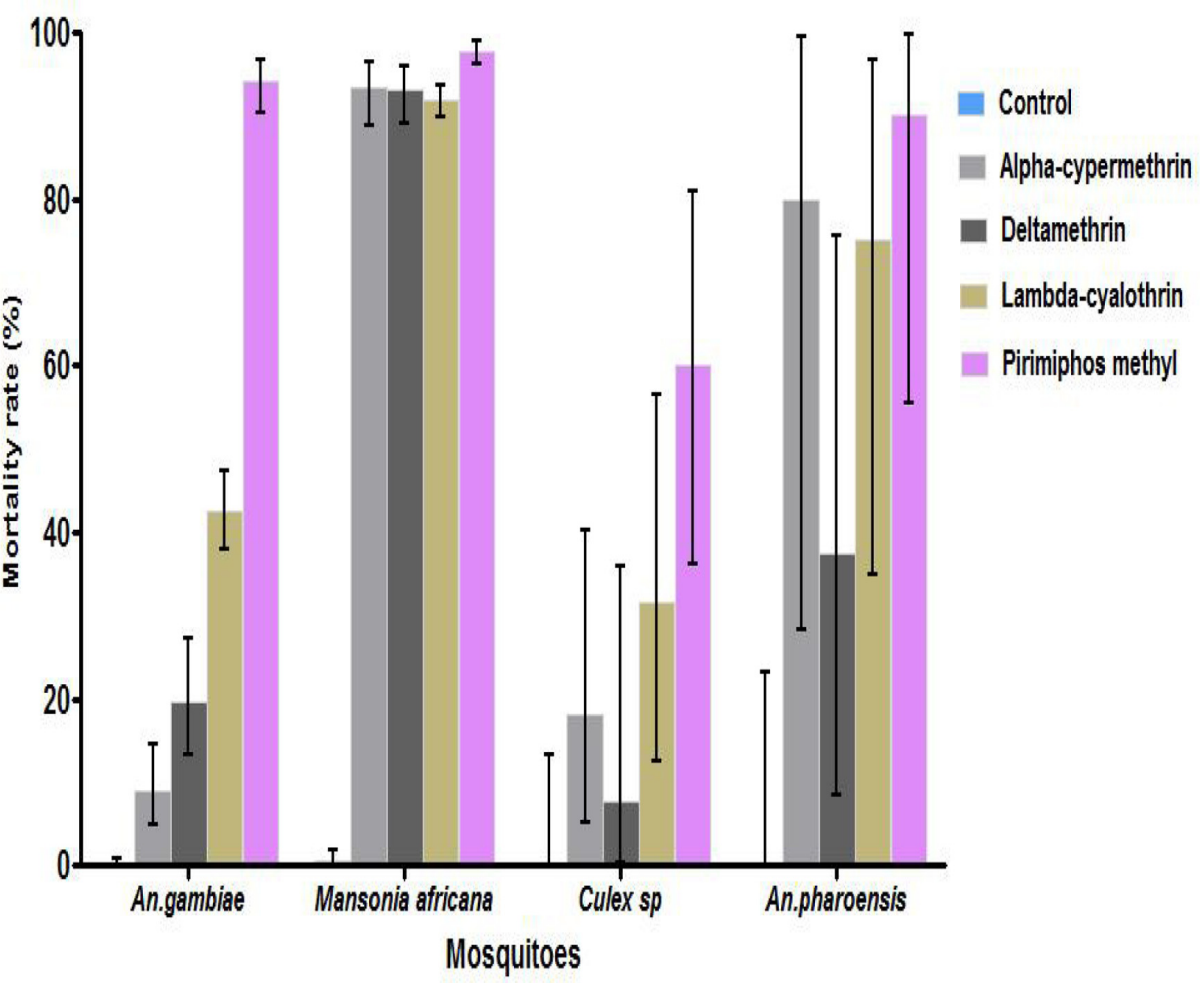
Cumulated mortality rates recorded with pyrethroids, compared to pirimiphos methyl toxicity during six months evaluation in semi-field conditions in Malanville. Control = untreated huts.

The toxicity or lethal property of pyrethroids used in IRS was significantly low with *An*. *gambiae s*.*l* (< 46%) but high for other predominant species such as *Mansonia africana* (93% to 97%) (Fig 3). Pirimiphos methyl induced similar mortality rates (≥ 94%) against *An*. *gambiae s*.*l* and *Mansonia africana*. *Culex spp* and *Anopheles pharoensis* collected were not represented enough in the study to sustain a powerful statistical analysis.

**Fig 4:**
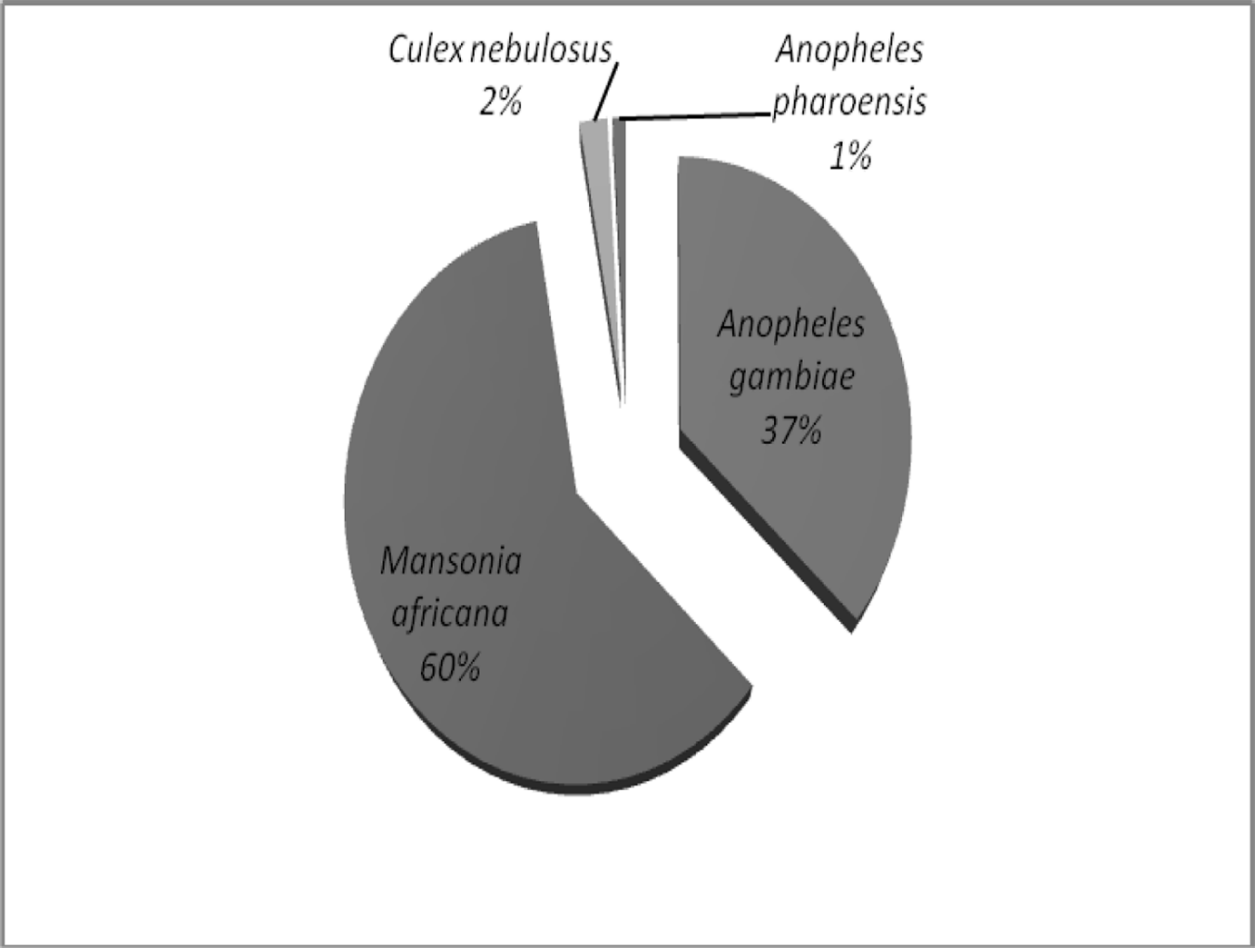
Repartition of mosquito’s species entering the experimental huts in Malanville. Females host-seeking mosquitoes collected during the six month evaluation period represent the species composition of mosquito.

*An*. *gambiae s*.*l* and *Mansonia africana* were the predominant species on the study site (Fig 4).

WHO susceptibility tests were performed with live adult mosquito species collected directly from the untreated hut. DDT, permethrin, lambda-cyhalothrin and deltamethrin induced a mortality rate of 50% for *Mansonia africana* at 66, 20, 14 and 12 minutes respectively suggesting that *Mansonia africana* was highly susceptible to pyrethroids. Permethrin, lambda-cyhalothrin and deltamethrin induced 50% mortality of *An*. *gambiae s*.*l* at 228, 128 and 94 minutes respectively (Table 2) indicating that adult *An*. *gambiae s*.*l* are highly resistant with KDT50 over a 60 min exposure time as recommended by the WHO susceptibility test.

**Table 2:** Induced KDT_50_ and _95_ by deltamethrin, permethrin, lambda-cyhalothrin and alphacypermethrin on wild adults of *Anopheles gambiae s*.*l* and *Mansonia africana* using quantitative WHO tests.

***Anopheles gambiae s*.*l***			
**Insecticides**	**KDT**	**T**	**CI-95%**
Deltamethrin	50	94^a^	[66.98–118.71]
Deltamethrin	95	243^x^	[214.58–277.65]
Permethrin	50	228^b^	[205.50–251.06]
Permethrin	95	376^y^	[344.99–418.09]
Lambda-cyalothrin	50	128^a^	[100.71–154.75]
Lambda-cyalothrin	95	277^x^	[245.46–316.53]
***Mansonia africana***			
Deltamethrin	50	12^a^	[5.12–18.48]
Deltamethrin	95	40^x^	[31.26–48.34]
DDT	50	66^b^	[59.08–72.43]
DDT	95	94^y^	[86.25–104.58]
Permethrin	50	20^a^	[11.86–27.66]
Permethrin	95	48^x^	[32.55–57.22]
Lambda-cyalothrin	50	14^a^	[7.53–22.77]
Lambda-cyalothrin	95	42^x^	[30.45–52.92]

**CI-95%**: 95% confidence interval, **T**: time for which 50% and 95% of exposed mosquitoes knock down; **KDT**: knock down time. The letters a and b are used for KDT 50 and x and y are used for KDT 95.

The letters a and x mean that there are no significant difference between the tested insecticides and the letter b and y mean that KDT is significantly higher.

### Cone test

The effectiveness of PermaNet® 2.0 (deltamethrin treated net) and OlyseNet® (permethrin treated net), which are widely used in Benin, were evaluated in the laboratory (phase I). Table 3 shows the efficacy of each net following the WHO cone bioassay. According to WHO [18], the threshold of bio-efficacy is 80% mortality or 95% knock-down in exposed mosquitoes. All nets recorded mortality rates of under 80% after 24 hours and less than 95% knock down after 60 minutes with resistant *An*. *gambiae s*.*l* from Malanville. Mortality rates were 23.71% for OlyseNet® and 39.06% for PermaNet®. However both nets induced 100% mortality with Kisumu (Table 3) suggesting a resistance effect with wild mosquitoes.

**Table 3:** Mortality and Knock-down (KD) rates of *An*.*gambiae* "KISUMU" and *An*.*gambiae s*.*l* from Malanville exposed to Olyset and Permanet 2.0 in cone bioassay.

Net	Strain	N tested	% KD	Mortality rate	CI-95%
**Olyset**	**Kisumu**	80	100	100	[96.97–100]
**Olyset**	**WT**	97	83.5	23.71	[15.66–33.43]
**Permanet 2.0**	**Kisumu**	79	100	100	[96.97–100]
**Permanet 2.0**	**WT**	64	91.4	39.06	[27.1–52.07]

**N**: number, **% KD**: percentage of knock down mosquito during exposition, **CI** -**95%**: 95% confidence interval which is referring to the mortality rate; **WT**: wild type *Anopheles gambiae s*.*l*.

#### Tunnel test

In the tunnel tests, 77.55% of wild type *An*. *gambiae s*.*l* were able to go through the untreated net to get in compartment B (Table 4). The penetration rate declined significantly with LLINs compared to the control (P<0.05). Less than 15% and 5% of the susceptible mosquitoes passed through the pierced PermaNet® and OlyseNet® respectively. Around 57.14% and 63.64% of the wild mosquitoes moved through the pierced PermaNet® and OlyseNet® respectively. Furthermore, the lethal action of PermaNet® and OlyseNet® was significantly higher (P<0.05) with Kisumu (respectively 92.73% and 79.74%) than with wild *An*. *gambiae s*.*l* populations (respectively 41.86% and 58.14%).

**Table 4:** The efficacy of Olyset and Permanet 2.0 against *An*.*gambiae s*.*l* collected from Malanville and laboratory susceptible ‘Kisumu’ strain in tunnel test.

Mosquito population	LLINs tested	Number tested	Penetrating rate	CI-95%	Blood feeding rate	CI-95%	%Immediate mortality	% Overall mortality	CI-95%
**Kisumu**	**UntreatedNet**	**49**	**77.55**	[**65.87–89.23**]	**79.59**	[68.31–90.88]	**0**	**0**	[0.0–7.53]
**Kisumu**	**Olyset**	**45**	**4.44**	[0.58–10.47]	**0**	[0.00–8.02]	**64.55**	**79.74**	[68–91.49]
**Kisumu**	**Permanet 2.0**	**47**	**14.89**	[4.72–25.07]	**0**	[0.00–7.13]	**80.6**	**92.73**	[85.30–100.15]
**WT *An*. *gambiae s*.*l***	**Olyset**	**49**	**57.14**	[43.29–71.00]	**79**	[65.31–90.87]	**48.84**	**58.14**	[44.33–71.95]
**WT *An*.*gambiae s*.*l***	**Permanet 2.0**	**55**	**63.64**	[50.92–76.35]	**89.09**	[80.85–97.33]	**32.56**	**41.86**	[28.05–55.67]

Parameters were calculated after 24h observation. **CI**-**95%**: 95% confidence interval. **WT**: Wild type.

Blood feeding rate means the proportion of blood fed mosquitoes in the total number of mosquitoes released into compartment A of the tunnel. The penetration rate is the proportion of mosquitoes recorded in compartment B among the total number released in compartment A. Mortality rate is the proportion of dead mosquito in the total mosquito released in compartment A.

## Discussion

The present study assessed repellency and toxicity of lambda-cyhalothrin, alpha-cypermethrin and deltamethrin in experimental huts and in the laboratory. The efficacy of Malaria vector control tools treated with pyrethroids has decreased when comparing their effect on wild and susceptible mosquitoes. Insecticide resistance might be the principal reason for this decrease.

Until 2004, malaria vector populations were fully susceptible to lambda-cyhalothrin and deltamethrin [2, 5]. This is the reason why CREC and Institut de Recherche pour le Développement (IRD) built their experimental huts in Malanville since then so as to conduct semi-field evaluation studies. All quantitative susceptibility tests undertaken showed high pyrethroids resistance in rice growing areas of northern Benin and although molecular analysis has not been performed in this study, the results are consistent with the resistance increase observed in 2011 in Malanville [6]. A review of literature over a period of five years showed an increase in L1014F Kdr frequency from 6% to 90% supporting the phenotypic resistance observed. In addition, resistance to deltamethrin, another insecticide in the family of pyrethroids, has emerged recently suggesting a spread of pyrethroid resistance across *Anopheles* populations [22, 23]. The finding of this study confirms a drastic decline in susceptibility to DDT and pyrethroids in the northern part of Benin as shown in previous investigations [22, 23]. Malanville being a highly agricultural area, the intense and uncontrolled use of pesticide in the rice field is frequent and is likely to have resulted in the selection pressure of Anopheles collected in the area in turn leading to resistance in these mosquitoes to pyrethroids [6].

While nets treated with pyrethroid are being constantly distributed nationwide based on WHO recommendation for net efficacy, our study indicated that these nets whether tested in controlled conditions or in semi-field conditions have low efficacy on the wild malaria vectors. Similar results were observed by N’Guessan et *al*. [7] in the semi-field assessment of LLINs in southern Benin.

A repellent effect was observed in controlled conditions in tunnel as very few mosquitoes of the susceptible strain were observed passing through the pierced barrier of the pyrethroids treated nets. This repellence though was reduced with the wild mosquitoes which fed easily on the bait. Otherwise, the treated nets would prevent the mosquitoes from Malanville from entering or prompted the early exit of these mosquitoes from exposed areas. This may not be specific to Anopheles or pyrethoids as Stanczyk et *al*., [24] has observed similar results with *Aedes aegypti* mosquitoes with DEET exposure.

The significant difference in toxicity for the mosquito strains (laboratory and wild) may be associated with resistance to pyrethroids. Furthermore, the lethal action of PermaNet® and OlyseNet® was significantly higher for the susceptible strain than with wild *An*. *gambiae s*.*l*. which suggests a correlation between increasing frequency of Kdr and decreasing mortality with consequences for insecticide resistance in *An*. *gambiae s*.*l*.

This study is the first to report the susceptibility of *Mansonia African* which is the second most predominant mosquitoes in our study area. Given that adult host seeking mosquitoes are fully susceptible to pyrethroids and DDT, even if vector control strategies using pyrethroids do not reduce malaria transmission significantly, through the toxicity and repellent effect these interventions would significantly contribute to reduce the burden due to *Mansonia africana*. Epidemiologically, this would dramatically reduce *Lymphatic filariasis* in Malanville as *Mansonia* is a potent vector of this neglected disease [25, 26].

The reduced efficacy of the pyrethroids nets observed in this study justifies the choice of the National Malaria Control Program (NMCP) of Benin as it encourages the use of insecticides families other than pyrethroids for malaria vector control strategies, especially for IRS implementation [27]. Recently, to prevent the spread of bendiocarb resistance [11], the NMCP used pyrimiphos methyl in mosaic with bendiocarb to manage pyrethroids and carbamates resistance in Atacora, northern Benin [16]. Several studies showed that agricultural practices select insecticide resistance [28, 29]. However, Sovi et *al*. [8] showed in a community evaluation, that there was no significant impact of resistance on behavior and on the effectiveness of LLINs and believed that continued malaria transmission is due to not only to malaria vector’s resistance but also environment factors.

To develop new generation of lasting and effective tools, it is important to consider, the problem of resistance. Malaria vector control tools must guaranty protection against mosquito populations irrespective of their resistance and susceptibility status. Obviously the survival of mosquitoes after a blood meal has significant epidemiological consequences as transmission of malaria occurs during this period. Even if mosquitoes successfully break through the excito-repellency barrier, they may not survive long enough to transmit malaria. It is argued that the excito-repellency effect and deterrence of mosquitoes may also attenuate or even reverse communal protection if it diverts mosquitoes to non-users rather than killing them outright [30].

Even though the use of insecticide treated materials may select resistance, it cannot be discouraged otherwise the transmission of malaria will increase. Efficacy of these tools in successfully controlling mosquito bites should constantly be monitored. New insecticide compounds should be introduced in area with low efficacy of pyrethroid and it is urgent to review strategies to reduce malaria transmission in high pyrethroids resistance areas. We suggest various resistance management approaches including the alternation of IRS with LLINs distribution. During the first two years, LLINS will be implemented and IRS will then be scaled up after 3 years. Nevertheless, pyrethroids are of great importance against mosquito bites in area endemic to Neglected Tropical Diseases such as *lymphatic filariasis*, highlighting the need for integrated vector control in a country such as Benin.

## Conclusion

A reduced efficacy of pyrethroids against wild *An*. *gambiae s*.*l* was noted in high insecticide resistance area in northerrn Benin. This study through a semi-field and laboratory evaluation demonstrated the reduced protective efficacy of pyrethroids-based LLINs and IRS. Therefore, it is crucial to find alternative class of insecticide with similar security use for human and the environment or to adopt targeted approaches for pyrethroids resistance management.

## Supporting Information

S1 Table Summary of results for host-seeking *An*.*gambiae s*.*l* in experimental huts (6 months data collected after treatment in Malanville). (DOC)
