# Supplementary material for: Impact of Insecticide Resistance on the Effectiveness of Pyrethroid-Based Malaria Vectors Control Tools in Benin: Decreased Toxicity and Repellent Effect
Source: PLoS One. 2015 Dec 16;10(12):e0145207. doi: 10.1371/journal.pone.0145207 (PMC4682945; doi:10.1371/journal.pone.0145207)
Supplement: S1 Table — (DOC) [file pone.0145207.s001.doc]

## Table: Summary of results obtained for host-seeking An.gambiae s.l in experimental huts (6 months data collected after treatment in Malanville)

| **Treatments** | **Month** | **Total females caught** | **Dterrency (%)** | **Exophily (%)** | | | **Blood feeding (%)** | | | | **immediate corrected mortality(%)** | | **overall corrected mortality (%)** | | |
| --- | --- | --- | --- | --- | --- | --- | --- | --- | --- | --- | --- | --- | --- | --- | --- |
|  |  |  |  | **Rate** | **95%Conf Lim** | **Induced exophily (%)** | **Rate** | **95%Conf lim** | **Blood feeding inhibition** | | **Rate** | **P** | **Rate** | **95%Conf lim** | **P** |
| **untreated hut** | 1 | 44 | - | 4,55 | [1,61-10,70] | - | 97,73 | [93,32-102,13] - | | | 0 | - | 0 | - | - |
|  | 2 | 49 | - | 12,24 | [3,07-21,42] | - | 67,35 | [54,22-80,48] | | - | 0 | - | 0 | - | - |
|  | 3 | 114 | - | 11,4 | [5,57-17,24] | - | 80,7 | [73,46-87,95] | | - | 0 | - | 0 | - | - |
|  | 4 | 125 | - | 10,4 | [5,05-15,75] | - | 82,4 | [75,72-89,08] | | - | 0 | - | 0 | - | - |
|  | 5 | 138 | - | 13,04 | [7,42-18,66] | - | 88,41 | [83,06-93,75] | | - | 0 | - | 0 | - | - |
|  | 6 | 136 | - | 13 | [7,40-18,64] | - | 88,41 | [83,06-93,75] | | - | 0 | - | 0 | - | - |
| **Lambda- Cyhalo** | 1 | 60 | 10,26 | 56,67 | [44,13-69,21] | 54,6 | 70 | [58,40-81,60] | | 28,37 | 7,91 | NS | 14,73 | [5,76-23,70] | 0,02 |
|  | 2 | 24 | 51,02 | 33,33 | [14,47-52,19] | 24,03 | 50 | [30,00-70,00] | | NS | 8,33 | 0,04 | 25 | [7,68-42,32] | P<0.001 |
|  | 3 | 73 | 35,96 | 32,88 | [22,10-43,65] | 24,24 | 65,75 | [54,87-76,64] | | 18,52 | 13,7 | P<0.001 | 24,66 | [14,77-34,55] | P<0.001 |
|  | 4 | 105 | 16 | 29,52 | [20,80-38,25] | 21,34 | 79,05 | [71,26-86,83] | | NS | 6,67 | P<0.001 | 14,29 | [7,59-20,98] | P<0.001 |
|  | 5 | 100 | 27,54 | 32 | [22,86-41,14] | 21,8 | 93 | [88-98] | | NS | 2 | NS | 9 | [3,39-14,61] | P<0.001 |
|  | 6 | 111 | 19,57 | 18,92 | [11,63-26,21] | NS | 93,69 | [89,17-98,22] | | NS | 1,8 | NS | 5,41 | [1,20-9,61] | 0,01 |
| **Delta** | 1 | 29 | 61,54 | 58,62 | [40,70-76,55] | 56,65 | 68,97 | [52,13-85,80] | | 29,43 | 25,9 | P<0.001 | 29,43 | [12,84-46,02] | P<0.001 |
|  | 2 | 36 | 26,53 | 55,56 | [39,32-71,79] | 49,35 | 58,33 | [42,23-74,44] | | NS | 0 | NS | 38,89 | [22,96-54,81] | P<0.001 |
|  | 3 | 102 | 10,53 | 38,24 | [28,80-47,67] | 30,29 | 85,29 | [78,42-92,17] | | NS | 3,92 | 0,03 | 35,29 | [26,02-44,57] | P<0.001 |
|  | 4 | 111 | 11,2 | 35,14 | [26,25-44,02] | 27,61 | 86,49 | [80,13-92,85] | | NS | 3,6 | 0,03 | 21,62 | [13,96-29,28] | P<0.001 |
|  | 5 | 93 | 32,61 | 34,41 | [24,75-44,06] | 24,57 | 95,7 | [91,58-99,82] | | NS | 3,23 | 0,03 | 6,45 | [1,46-11,44] | P<0.001 |
|  | 6 | 100 | 27,54 | 24 | [15,63-32,37] | 12,6 | 96 | [92,16-99,84] | | NS | 3 | 0,04 | 5 | [0,73-9,27] | 0,01 |
| **Alpha-Cyp** | 1 | 37 | 61,54 | 40,54 | [24,72-56,35] | 37,71 | 86,49 | [75,47-97,50] | | NS | 8,74 | NS | 11,5 | [1,22-21,78] | 0,05 |
|  | 2 | 26 | 46,94 | 42,31 | [23,32-61,30] | 34,26 | 46,15 | [26,99-65,32] | | NS | 19,23 | P<0.001 | 30,77 | [13,03-48,51] | P<0.001 |
|  | 3 | 70 | 38,6 | 37,14 | [25,82-48,46] | 29,05 | 68,57 | [57,70-79,45] | | NS | 18,57 | P<0.001 | 24,29 | [14,24-34,33] | P<0.001 |
|  | 4 | 100 | 20 | 31 | [21,94-40,06] | 22,99 | 85 | [78-92] | | NS | 6 | 0,01 | 11 | [4,87-17,13] | P<0.001 |
|  | 5 | 108 | 21,74 | 35,19 | [26,18-44,19] | 25,46 | 93,52 | [88,88-98,16] | | NS | 2,78 | 0,05 | 6,48 | [1,84-11,12] | P<0.001 |
|  | 6 | 96 | 30,43 | 27,08 | [18,19-35,97] | 16,15 | 92,71 | [87,51-97,91] | | NS | 3,13 | 0,04 | 6,25 | [1,41-11,09] | P<0.001 |
|  |  |  |  |  |  |  |  |  |  |  |  |  |  |  |  |

Lambda Cyhalo=Lambda-Cyhalothrin; Delta= Deltamethrin; Alpha Cyper= Alpha-cypermethrin; 95%Conf lim= 95% confidence limite, NS = not significant and '-' = reference rate.
